# Near Infrared Spectroscopy Facilitates Rapid Identification of Both Young and Mature Amazonian Tree Species

**DOI:** 10.1371/journal.pone.0134521

**Published:** 2015-08-27

**Authors:** Carla Lang, Flávia Regina Capellotto Costa, José Luís Campana Camargo, Flávia Machado Durgante, Alberto Vicentini

**Affiliations:** 1 Graduate Program in Botany, Instituto Nacional de Pesquisas da Amazonia, Manaus, Brazil; 2 Department of Biodiversity, Instituto Nacional de Pesquisas da Amazonia, Manaus, Brazil; 3 Biological Dynamics of Forest Fragments Project (INPA/STRI), Manaus, Brazil; 4 Department of Environmental Dynamics, Instituto Nacional de Pesquisas da Amazonia, Manaus, Brazil; Technical University in Zvolen, SLOVAKIA

## Abstract

Precise identification of plant species requires a high level of knowledge by taxonomists and presence of reproductive material. This represents a major limitation for those working with seedlings and juveniles, which differ morphologically from adults and do not bear reproductive structures. Near-infrared spectroscopy (FT-NIR) has previously been shown to be effective in species discrimination of adult plants, so if young and adults have a similar spectral signature, discriminant functions based on FT-NIR spectra of adults can be used to identify leaves from young plants. We tested this with a sample of 419 plants in 13 Amazonian species from the genera *Protium* and *Crepidospermum* (Burseraceae). We obtained 12 spectral readings per plant, from adaxial and abaxial surfaces of dried leaves, and compared the rate of correct predictions of species with discriminant functions for different combinations of readings. We showed that the best models for predicting species in early developmental stages are those containing spectral data from both young and adult plants (98% correct predictions of external samples), but even using only adult spectra it is still possible to attain good levels of identification of young. We obtained an average of 75% correct identifications of young plants by discriminant equations based only on adults, when the most informative wavelengths were selected. Most species were accurately predicted (75–100% correct identifications), and only three had poor predictions (27–60%). These results were obtained despite the fact that spectra of young individuals were distinct from those of adults when species were analyzed individually. We concluded that FT-NIR has a high potential in the identification of species even at different ontogenetic stages, and that young plants can be identified based on spectra of adults with reasonable confidence.

## Introduction

Species identification requires a high level of taxonomic expertise, confirmation by specialists [1] and the presence of reproductive materials [2], factors that hinder accurate identification of plants in areas with high biological diversity as in the tropics. Using the morphology of dried plant specimens in a non-reproductive state to define species can generate a high rate of misidentification [3], yet is a frequent procedure in forest inventories. The classic use of morphological traits for species identification has several limitations, including phenotypic plasticity and the existence of cryptic taxa. The identification of sterile samples of young plants is most often not possible with conventional identification keys, since morphology-based plant keys are almost always designed for reproductive adult material [4]. This represents a major limitation for those studying seedlings and juveniles, which have been generally little studied for morphology. The need for tools to identify species at all developmental stages has long-been emphasized, but there is still much to do to expand the taxonomic knowledge of plants in the early stages of life, especially in areas like the Amazon basin [5]. However, emerging technologies can help, providing effective and accurate means to identify species at any of their developmental stages.

The identification of taxa using a standardized region of DNA (DNA barcoding) [6] has received attention recently, and is being developed by an international initiative. Molecular data have been very useful in solving problems of discrimination of species [7, 8]. Kress et al. [9] showed that a barcode containing three DNA loci was effective in identifying well-defined tree species on Barro Colorado Island, achieving 98% correct identifications. However, for highly diverse environments, such as the Amazon, DNA barcoding, in its current form, has still low predictive power for species, even when a larger number of markers are used, because many species are of recent origin and many genera do not form monophyletic groups [10]. For closely related species, genetic and morphological differentiation is difficult and the assignment of a species is ambiguous [11]. Furthermore, the method of DNA barcoding is still expensive for large-scale applications in hyperdiverse areas [12]. Since knowledge of the chemical and structural basis underlying the distinctive signal between species is still lacking, FT-NIR can not substitute molecular analysis for systematics, which should be based on genetic differences. However, easy and fast species identification for variety of ecological and others types of studies, is the greatest promise of FT-NIR and others related spectral techniques.

Morphological and molecular data are similar in several points: they can be based on sterile material, use advanced technology, generate a lot of data and need taxonomic expertise to be calibrated. Near infrared spectroscopy (FT-NIR) is an alternative method that is highly cost-effective, quick, non-destructive, and requires no pretreatment of samples [13]. The cost of a good FT-NIR spectrometer, which can process an unlimited number of samples, is about the same of DNA barcoding ~ 800 samples, and the only maintenance cost is the exchange of lamps, and general equipment maintenance.

The FT-NIR can detect with high accuracy any molecule in which the principal chemical bonds are CH, OH, NH, SH or C = O [14, 15]. When an organic sample is irradiated, chemical bonds continuously vibrate causing a wave motion that is characteristic of that functional group [16]. The contact of the incident FT-NIR light on leaf tissue generates a spectral response that is a function of the chemical composition and structure of cells and internal morphology of the leaf [17], which may be characteristic of the species.

Applications of FT-NIR as a tool for the analysis of chemical and physical properties can be found in virtually all areas of science. In wood science, FT-NIR has been used to predict the physical, mechanical and chemical properties of wood [18, 19, 20, 21]. There are also applications for determining the geographical origin of individual plants [14], and in plant taxonomy, recent studies that have used the technique have achieved successful identification rates ranging from 80 to 100% [22, 23, 24, 25]. Other spectroscopic techniques based on different bands of infrared radiation have been used to identify species or their chemical properties [26].

Spectroscopy has, therefore, been revealed as a promising tool in the discrimination and identification of adult individuals of plant species. This encouraged us to ask if young and adult plants of the same species had similar spectral signature, and, if so, whether FT-NIR spectra of well-identified adults could therefore be used to predict the species identity of young plants. The ability to identify saplings and seedlings reliably and swiftly would open many avenues for studies of demographics, life history, dispersal, and others areas that are currently limited by poor knowledge of developmental changes in the morphology of plant species.

## Materials and Methods

Leaf samples used in this study came from two areas close to the city of Manaus, Amazonas, Brazil: the Ducke Forest Reserve (2°55'S, 59°59'W) and an area within the Biological Dynamics of Forest Fragments research project (BDFFP) (2°30'S, 60°W), located ca. 50 km N of Ducke Reserve. Both reserves are covered by dense lowland Amazonian rainforest [27]. Ducke and Biological Dynamics of Forest Fragments research project (BDFFP) are under the jurisdiction of the Brazilian National Institute for Amazon Research (INPA), which issued permits for the sampling involved in the present study.

### Sample collection

The botanical material used was from herbarium specimens collected in Ducke Reserve from January to March 2013 (8.38% of specimens), and herbarium specimens that were collected from trees tagged at permanent plots belonging the BDFFP project over the past 35 years (91.6%). They represent the developmental stages of seedlings, juveniles and adults of the species concerned. All leaf samples, both the newly collected and the BDFFP ones, were not preserved in alcohol in the field; these leaves were prepared following the normal procedures used for herbarium specimens, oven-dried (60°C) for 1 or 2 days until completely dry. Fruit for the production of seedlings and voucher material for production of dried specimens were collected from the Ducke Reserve. Fruits were processed to obtain seeds, which were germinated in a greenhouse. All seedlings produced were dried by the same process describe above. As germination and seedling production rates were very low for most species, it became necessary to supplement these by previously collected and identified herbarium specimens of seedlings. Most adult samples were identified by Douglas Daly of the New York Botanical Garden, an expert in Burseraceae. Identification of seedlings and juvenile samples was reviewed by Paul Fine of the University of Berkeley, California.

The species used in the study belong to the Burseraceae, a family known for the difficulties encountered when attempting species identification based solely on morphological characters. This is notable in the species-rich genus *Protium*, where it is especially difficult to identify seedlings and juveniles. A total of 346 specimens were used, 196 specimens from the adult stage and 150 at the juvenile or seedling stage. These represent 13 species of Burseraceae, 12 from the genus *Protium*, and one from the genus *Crepidospermum* (Table 1). We considered two forms of *Protium hebetatum* DC Daly, empirically distinct but not formally named. These are "form A" (hairier and with more bulbous leaflets) and "Form B" (slender glabrous leaflets and lightly haired petioles) (A. Andrade. comm.). We used a minimum number of 10 individuals per species.

**Table 1:** Number of specimens used to obtain FT-NIR spectra.

Species	Seedlings/Juveniles	Mature
***Protium apiculatum* Swart**	12	24
***Protium decandrum* (Aubl.) Marchand**	10	13
***Protium grandifolium* Engl.**	7	10
***Protium hebetatum* D.C. Daly forma A**	20	19
***Protium hebetatum* D.C. Daly forma B**	19	16
***Protium krukoffi* Swart**	18	15
***Protium occultum* Daly**	10	20
***Protium pallidum* Cuatrec.**	9	15
***Protium paniculatum* (Engl.) *var. nova***	12	13
***Protium paniculatum var*. *riedelianum* (Engl.) Daly**	8	13
***Protium sagotianum* Marchand**	11	16
***Protium subserratum* (Engl.) Engl.**	9	10
***Crepidospermum rhoifolium* (Benth.) Triana & Planch**	5	12
**Total**	**150**	**196**

### FT-NIR spectroscopy measurements

A total of 12 FT-NIR spectral readings were obtained for each specimen. We used three whole dried leaves per voucher specimen. Four spectral readings were collected per leaf, with two readings each on the adaxial (upper) and abaxial (lower) surfaces of the leaf (a total of 12 readings per plant). The reading points included the base and the apex of the respective sides of the leaf. This data were obtained for 346 samples from both Ducke Reserve and BDFFP. Leaf spectra were collected with a Thermo Nicollet spectrophotometer, using the Antaris FT-NIR II Method Development System (MDS) [28]. The spectral readings are expressed as absorbance values between the wavelengths 1000 to 2500 nm in the near-infrared and each spectrum consists of 1557 absorbance values. Each reading produced by the instrument was the average of 16 scans at a wavelength, a resolution of 8 cm^-1^. A black body was placed over the point where the spectral readings were collected to avoid light scattering. A background calibration reading was performed before each reading was taken.

### Analyses

The analyses were performed using two sets of data, (1) the absorbance at all wavelengths over the entire FT-NIR spectrum (1000 to 2500nm), a total of 1557 wavelengths, and (2) a selection of the most informative wavelengths.

The most informative wavelengths were selected by discriminant analysis. The procedure aimed to capture the set of independent variables that best predicted species identity, since not all of the spectra are necessarily informative for discrimination. In spectral data, it is common that some spectral regions vary in a consistent fashion between samples to be discriminated, while others do not vary (and therefore are uninformative) and some vary inconsistently, providing only noise. Cleaning this noise may increase the discrimination power. We used a stepwise method to select the wavelengths, involving a process of inclusion and exclusion of independent wavelengths in the discriminant function one at a time, based on their discriminatory power. To do this we used the stepclass function of the Klar package [29], where the process is completed when all independent wavelengths are included in the function or the wavelengths excluded are judged not to contribute significantly to the discrimination [30]. However, because of the large number of wavelengths of the spectrum (1557), the maximum number of selected wavelengths was defined as one-third of the number of samples analyzed, following the discriminant analysis premise of Williams and Titus [31].

Discriminant functions (LDA) were generated to assess the capacity of the spectra to distinguish species, regardless of developmental stage. Discriminant analyses were conducted using either absorbance values at all wavelengths of the FT-NIR (1000–2500 nm) spectrum, or those wavelengths determined by the stepwise method to be the most informative. The functions were generated from four different sets of leaf data readings: 1) average of 12 readings (abaxial+adaxial); 2) the average of six adaxial readings; 3) the average of six abaxial readings, and 4) using one reading randomly selected from the 12 taken per individual (Table 2).

**Table 2:** Description of tests based on FT-NIR spectra to discriminate between Burseraceae species.

		Linear Discriminant Analyses (LDAs) Models		
	Dataset	Adult Model	Young Model	Combined Model
Mean of readings	Adaxial+Abaxial	x	x	x
	Adaxial	x	x	x
	Abaxial	x	x	x
	Single spectrum	x	x	

Model Adult, function generated with samples of adults only. Validation: samples of seedlings and young plants; Young Model, function generated with samples of seedlings/young only. Validation: samples of adults; Combined Model, function generated with 2/3 of the total sample, all developmental stages combined. Validation: the remaining 1/3 of the total sample. All listed combinations were tested both with all wavelength components, as with those identified by stepwise analysis as the most informative.

For each of these data sets, a function was generated using only data from adult plants (Adult Model). The external samples of seedlings/juveniles were used to validate the model. The converse process was carried out where the equation was constructed based on data from juvenile plants (Young Model), validated with external samples from adults. As a further test, a function was generated with 2/3 of samples including both young and adults (Combined Model), and using the remaining 1/3 for validation. Each model was repeated 100 times with randomization of the subassemblies and the average of the 100 iterations was compared.

Finally, we evaluated the capacity of the spectral data to discriminate development stages. For this model the species identity was suppressed, and the samples divided only into young and adult groups. The model was built with 2/3 of the data and validated with the remaining third.

For each of the tests described, we obtained the percentage of correct identifications. Analyses were performed in R 2.10.0 environment [32].

## Results

### Is it possible to predict the identity of young based on adults?

#### Tests using all wavelengths of the FT-NIR spectrum (1000 to 2500nm)

**Fig 1:**
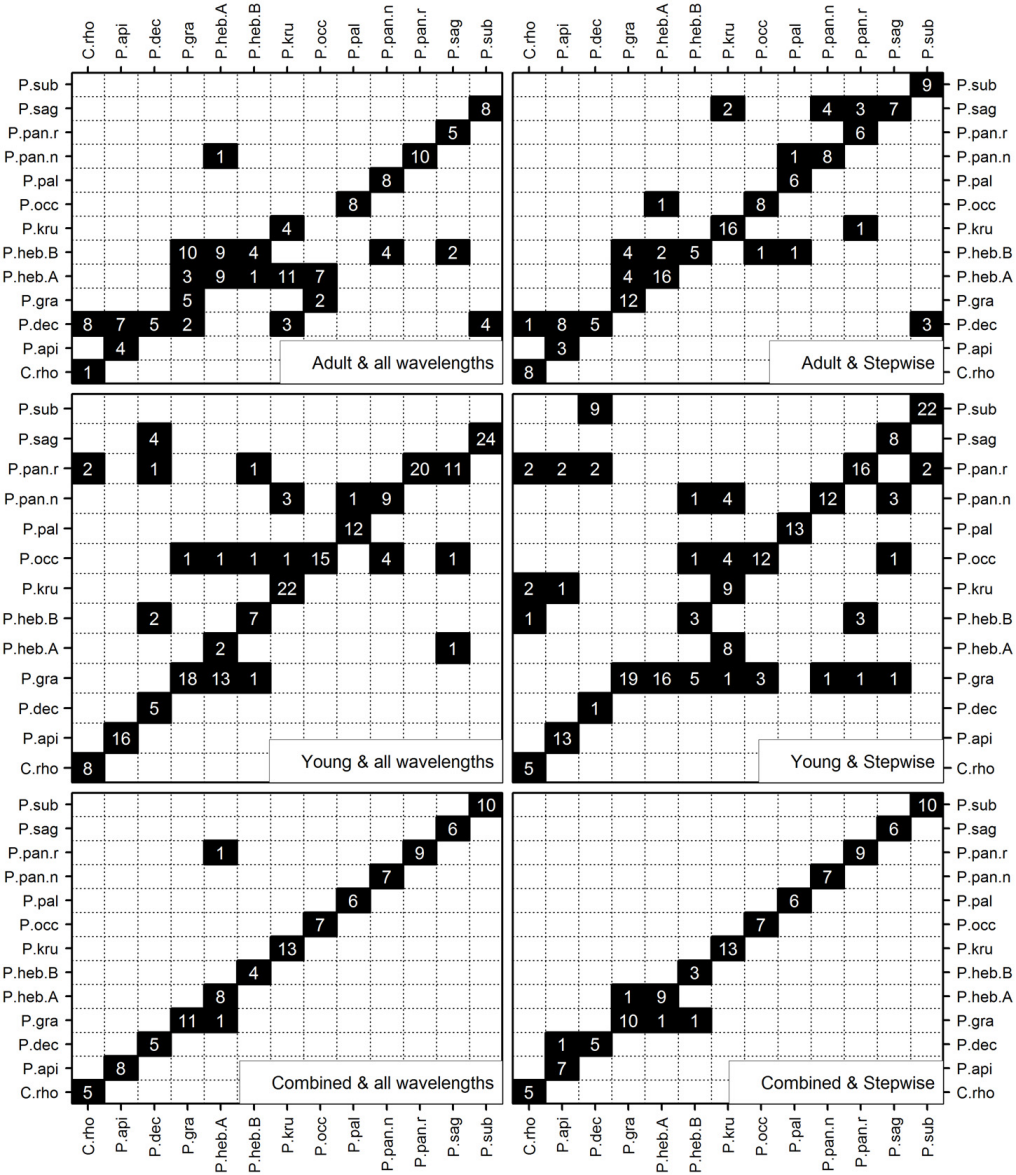
Matrices with the results of Discriminant Analysis for the three models (Adult, Young and Combined) and using average of 12 readings (abaxial+adaxial), and for both all wavelengths (left) and stepwise selected wavelengths (right). The observed species names are given in columns, while predicted names are given in rows. Therefore, the values on the diagonal are correct predictions, and off-diagonal values are wrong predictions. Abbreviations: C. rho = *Crepidospermum rhoifolium*; P. api = *Protium apiculatum*; P. dec = *Protium decandrum*; P. gra = *Protium grandifolium*; P. heb.A = *Protium hebetatum* forma A; P. heb. B = *Protium hebetatum* forma B; P. kru = *Protium krukoffi*; P. occ = *Protium occultum*; P. pal = *Protium pallidum*; P. pan.n = *Protium paniculatum var*. *nova*; P.pan.r = *Protium paniculatum var*. *reidelianum*; P. sag = *Protium sagotianum*; P. sub = *Protium subserratum*.

The results of the test series are shown in Table 3 and Fig 1. When using all FT-NIR wavelengths the models built with spectroscopic readings from adults were able to predict the identity of young in 48 to 57% of occasions, averaged over all species. The model constructed with samples of young plants, was able to predict the identity of adults with 75–76% accuracy. For the model combining adult and young individuals, the average probability of success was 98% for the three sets of data tested.

**Table 3:** Results of discriminat analysis using the average of the readings and all wavelengths of the spectrum FT-NIR.

	Adult Model				Young Model				Combined Model		
	Dataset				Dataset				Dataset		
Species	MeanAd+Ab	MeanAd	MeanAb	Single Spectrum	MeanAd+Ab	MeanAd	MeanAb	Single Spectrum	MeanAd+Ab	MeanAd	MeanAb
*Protium apiculatum* Swart	66	50	59	54	100	100	100	96	100	100	100
*Protium decandrum* (Aubl.) Marchand	71	87	71	86	0	8	31	65	100	100	100
*Protium grandifolium* Engl.	80	100	100	100	70	50	70	20	100	100	100
*Protium hebetatum* D.C. Daly forma A	25	15	30	12	95	100	100	95	100	91	91
*Protium hebetatum* D.C. Daly forma B	47	57	21	53	12	0	19	12	98	98	90
*Protium krukoffi* Swart	22	55	94	47	85	77	73	76	100	100	100
*Protium occultum* Daly	100	100	90	98	100	95	95	100	100	100	100
*Protium pallidum* Cuatrec.	0	0	0	0	100	100	100	80	100	100	100
*Protium paniculatum* (Engl.) *var*. *nova*	66	100	58	27	69	92	69	100	100	100	100
*Protium paniculatum var*. *riedelianum* (Engl.) Daly	100	100	100	100	92	100	100	73	100	100	100
*Protium sagotianum* Marchand	36	36	45	64	100	87	87	86	100	100	100
*Protium subserratum* (Engl.) Engl.	11	33	33	11	80	70	80	70	100	100	100
*Crepidospermum rhoifolium* (Benth.) Triana & Planch	100	100	100	100	41	58	33	75	100	100	100
**Percentage of correct identification**	**48**	**57**	**56**	**51**	**76**	**75**	**76**	**75**	**98.6**	**98.4**	**98.6**

Mean Ad +Ab, the average of 12 readings; Mean Ad, the average of the readings of the adaxial surface; Mean Ab, the average of the readings abaxial surface; Single reading, a randomly selected reading. Hit percentage for each species and average percentage for models generated with each data set.

The average absorbance values of adaxial leaf surface provided better predictions of young´s identity from models based on adults than the abaxial surface. However, in general, all models based on the complete set of wavelengths from adults poorly predicted (average of 53% accuracy over all models and species) the identity of young individuals. Only three species (23%) achieved 100% accuracy when we used the averages of 12 readings, and only 5 species (38.4%) when we used the averaged readings from the adaxial surface. In *Protium pallidum* no accurate identification of young specimens was obtained, while all individuals were correctly identified at the adult stage. Two species (*Protium occultum* and *P*. *paniculatum* var. *riedelianum*) were consistently predicted correctly in both developmental stages. In general, using all wavelengths, the species were predicted correctly in only one phase of development, or when young using the model based on adults, or when adult using the model based on the spectral properties of young plants.

Using only one reading from randomly selected individual reading reduced predictive ability. The model based on measurements from adults to predict species identities in young plants had a similar level of success to those already obtained with the 12 readings average (51% accuracy). This also occurred with the Young Model, where the correct hits average was 75%. For most species, the percentage of correct responses with a randomly selected reading remained similar to the results previously obtained with the average of 12 readings.

#### Tests using the most informative, stepwise-selected, wavelengths

The results of this test series are shown in Table 4 and Fig 1. Twenty variables with the highest discriminating power were selected between the wavelengths 2072.53 to 2436.77 nm, from the initial 1557 measurements. Selection was made irrespective of developmental stage. The ability to predict species identity of young individuals using the Adult Model improved in the three data sets of averaged readings, increasing from 48–57% to 60–75% correct identifications. Most species were well predicted (75–100% correct identifications), and only three had poor predictions (27–60%). The predictions of adult identities based on spectroscopic responses of young plants decreased from 75–76% to 61–64% across all models, and the same occurred for the model combining young and adult plants.

**Table 4:** Results of discriminant analysis based on the average of 12 readings and the most informative wavelengths selected by stepwise modelling.

	Adult Model				Young Model				Combined Model		
	Dataset				Dataset				Dataset		
Species	MeanAd+Ab	MeanAd	MeanAb	Single Spectrum	MeanAd+Ab	MeanAd	MeanAb	Single Spectrum	MeanAd+Ab	MeanAd	MeanAb
*Protium apiculatum* Swart	75	100	33	67	91	96	83	96	100	100	100
*Protium decandrum* (Aubl.) Marchand	100	100	100	93	61	54	69	0	100	100	100
*Protium grandifolium* Engl.	100	100	100	90	30	20	30	70	87	100	75
*Protium hebetatum* D.C. Daly forma A	60	20	65	29	100	100	95	92	91	77	71
*Protium hebetatum* D.C. Daly forma B	84	68	79	47	0	0	6,0	53	60	70	41
*Protium krukoffi* Swart	89	83	89	16	35	40	35	69	100	100	100
*Protium occultum* Daly	60	50	100	100	80	70	95	100	100	100	100
*Protium pallidum* Cuatrec.	89	11	55	0	80	60	87	80	100	100	100
*Protium paniculatum* (Engl.) *var*. *nova*	67	58	92	11	92	85	54	84	100	100	86
*Protium paniculatum var*. *riedelianum* (Engl.) Daly	75	0	100	98	100	100	100	84	100	100	100
*Protium sagotianum* Marchand	27	36	18	45	81	75	75	68	100	87	87
*Protium subserratum* (Engl.) Engl.	89	100	67	44	50	80	10	30	100	100	100
*Crepidospermum rhoifolium* (Benth.) Triana & Planch	100	100	100	100	80	80	80	43	100	100	100
**Percentage of correct identification**	**75**	**60**	**73**	**52**	**64**	**58**	**61**	**73**	**97**	**94**	**96**

Mean Ad+Ab, the average of 12 readings; Mean Ad. the average of the readings adaxial surface (Ad); Mean Ab, the average of the readings adaxial surface; Single Reading, a randomly selected reading. Hit percentage for each species and average percentage for models generated with each set of data are given.

The model for prediction of the identity of young based on adult spectra with best results was that using the average of 12 readings (Ad + Ab). This had an average of 75% of correct species identifications of young plants. Only one species (*P*. *sagotianum*) had less than 60% of correct identifications, and the majority (nine species) had 75% or more correct responses.

For a randomly selected spectral reading, stepwise analysis retained 39 wavelengths between 1002.60 to 2495.41 nm as being the most informative. For most models there was no significant differences between this and the results obtained previously with all FT-NIR spectrum wavelengths (1000 to 2500 nm).

### Do young and adult plants differ in their NIR spectra?

**Fig 2:**
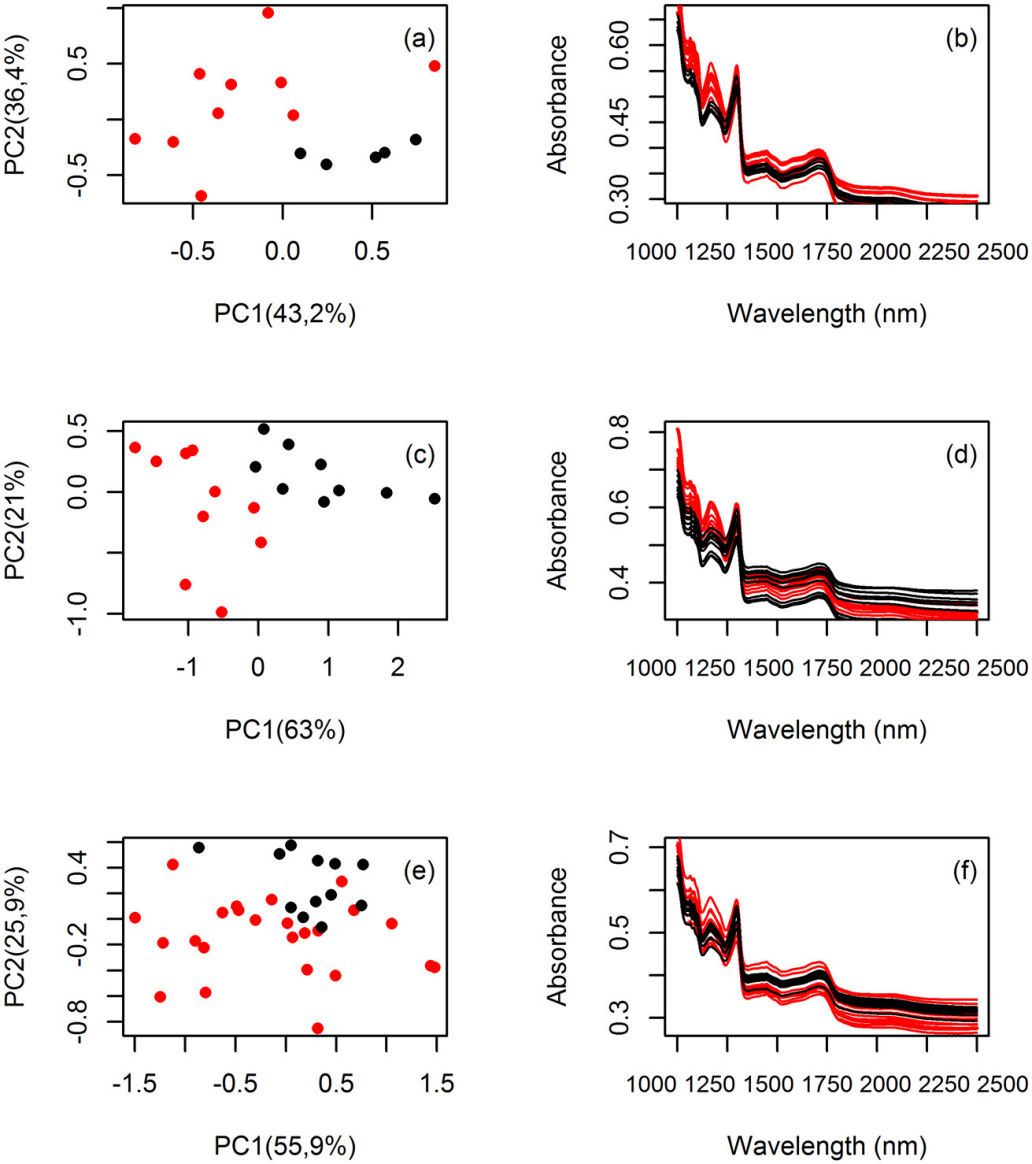
(a, c, e) Graphic representation of a two dimensional Principal Component Analysis (PCA) of FT-NIR spectra of young and adult individuals. (b, d, f) Representation of full spectra of individuals. Spectra is composed of 1557 wavelengths, for the average of 12 readings per individual. Young plants in black, adult plants in red. (a, b) *Protium grandifolium*; (c, d) *Protium subserratum* (e, f) *Protium apiculatum*.

When testing the capacity of near-infrared spectroscopy to discriminate between developmental stages, independent of species, discriminant analysis had an average accuracy of 99.9%, indicating that developmental stages consistently differ in their spectral signatures. Also within species the difference in spectral values between young and adult plants can be readily seen, both for the full spectra and for an ordination in two dimensions (Fig 2).

## Discussion

Our results indicated that near-infrared spectroscopy (FT-NIR) is an effective tool for discriminating tree species at different stages of development. In our study, we indicated that the best models for predicting species in early developmental stages are those containing spectral data from both young and adult plants, but even using only adult spectra is still possible to attain good levels of identification of young. And finally, we have demonstrated that young and adults of the same amazon trees species clearly differ in their spectra.

### Can young be identified based only on spectra from adults?

In this study we have shown that it is possible to identify young tree species based on spectral signatures of adults of the same species, although the level of accuracy obtained is still not as high as observed when identifications are made within the same developmental stage, or when both dvelopmental stages are used to produce the models. We obtained an all-species average of 75% correct identifications for young plants when discriminant equations were constructed with the most informative wavelengths. Comparing these results with rates of correct identifications even within the same developmental stage obtained by traditional methods (40–50% [3, 33]) or barcoding (70%, [34]), we conclude that the cost-benefit of spectroscopy-based method is higher, given its speed, low cost and ability to achieve successful hits. We are not advocating that these methods should be abandoned, but that FT-NIR can provide quick answers that will make possible a large expansion of plant ecology studies.

We expected that a random reading per individual would provide more accurate predictions, given the chance that several readings from the same leaf could contain local contamination, epiphylls, that might spoil the spectral pattern of a species. However, there was no improvement in hit rate with this procedure, and it seems that use of the an average of readings is a more effective way of smoothing possible local variations between leaves. Wavelength selection indicated the most informative region of the FT-NIR spectrum lay between 2000 and 2500 nm. A similar range (1666.66 to 2500 nm) was reported by Durgante et al. [22]. This region is related to the presence of carbohydrates such as cellulose, lignin and polysaccharides [35], compounds that are associated with the structure of plant cell wall [36]. As as whole, we suggest that the best protocol for building a wide range model for species discrimination based on FT-NIR spectra is to use an average of readings from both adaxial and abaxial leaf surfaces of each specimen, and the 2000–2500 nm region of the spectra.

Our study indicated that the best models for predicting species in early developmental stages are those that contain spectral data for young and adult plants. Our best result gave a 98.6% correct identification of the species, regardless of developmental stage, when we used the averaged readings and the whole FT-NIR spectrum (1000–2500 nm) for the model generated with samples of adults and young plants. Early studies of plants using diffuse reflectance measurements recognized that the plant cuticle and the underlying cell wall are the principal cause of spectral features [37, 38]. Castro-Esau et al. [39] demonstrate that leaves of the same species, but of different ages or health, will vary widely in their spectral reflectance properties, and showed that internal leaf structure influences leaf reflectance in the near-infrared region. From this we can infer that discriminant function models containing samples of young plants can better capture all possible spectral variability, both chemical or structural, between young and adults, and these models will be more efficient in predicting species identity at early stages of development. However, even in the absence of samples of young plants in the model, it is still possible to predict their identity based on adults, with a slightly higher degree of uncertainty, as demonstrated above. Because it is difficult and long term work to obtain samples of well identified young plants for building combined models, our results showing the possibility of using only adults as a base for models should provide the capacity to attain a higher level of identification for seedlings than we currently have.

These results are comparable to previous studies using FT-NIR for botanical taxonomy purposes. However, we have demonstrated for the first time that the spectral signatures of different developmental stages can be used for species determination. Previous work has achieved 100% accuracy in discriminating leaves *Eucalyptus globulus* Labill and *Eucalyptus nitens* Maiden [25] and rates ranging 84–92% with species of *Ephedra* in FT-NIRs [24], while Durgante et al. [22] achieved a success rate exceeding 96% for correct identifications of 10 Amazonian species from the family Lecythidaceae. Maree & Viljoen [40] indicated that both FT-NIR and FT-MIR spectroscopy (near and medium infra-red, respectively) can be used to discriminate between closely related plant species.

As seen, the spectroscopy FT- NIR may offer advantages such as reduced laboratory time and non-destructive sampling relative to other methods [16]. In highly diverse areas, such as Amazon rainforest, the FT-NIR appears as a highly reliable method that can provide more accurate measurements of local diversity [41, 42]. However, leaf FT-NIR spectral data for taxonomic purposes have some limitations, related to equipment and databasing. It is still unclear whether FT-NIR leaf spectra obtained with different instruments are comparable and mainly, the use of FT-NIR leaf spectra requires the structuring of repositories, which can be used to dynamically generate spectral signatures that will change as data are accumulated, and thus be used for species identification [22].

### Potential causes of the spectral differences between youth and adults

Our study found that young and adult plants clearly differ in their spectra, the hit rate on the separation of adults and juveniles beings 99.9%, regardless of the species. We can infer that it is possible to predict the developmental stage of a sample without necessarily knowing the species to which it belongs, at least for tropical tree species. Although there are spectral differences between the different stages of the same species, the difference in spectral signatures between species is large enough to allow the determination of species spectra using a different developmental stage of the sample tested.

Leaves of the trees change throughout the development, and these chemical, morphological and structural changes, may cause spectral differences between young and adult. It is well known that the size, leaf shape [43, 44], phenology [45], photosynthesis and water-use strategies [46] change along the developmental process. As a result, throughout a plant´s development, profound changes can be observed in leaf structure. Generally leaves of young individuals are more pliable, translucent and the structures of the leaf blade are still developing [47]. The leaves of seedlings and juveniles are also thinner and have lower concentrations of nitrogen, cellulose, hemicellulose and lignin than leaves of mature individuals [48]. Changes in terpene composition associated with the ontogenetic development have been also documented in leaves [49]. Coley and Barone [50] showed, for tropical species, that young leaves suffer high rates of herbivory, they exhibit an even greater diversity of defenses than mature leaves. They can protect themselves with a battery of secondary metabolites, often having higher concentrations, as well as compounds not found in the mature leaves [51, 52].

Leaves are complex assemblies of organic compounds and, because of this, may be expected to exhibit different spectral responses. Durgante et al. [22] reported that young and mature leaves of *Eschweilera amazoniciformis* had different spectral signatures, and consequently that predictions using spectra from mature leaves mostly resulted in incorrect identifications of species for young leaves. Abasolo et al. [53] used FT-NIR spectroscopy to discriminate hybrid *Corymbia* seedlings 4 and 8 months older, and found more reliable results for older rather than younger seedlings, attributing this difference to chemical changes that occurred during the four month developmental interval between groups.

The differences in spectroscopic properties of leaves of different stages might well be due to changes in cell wall composition, such as proportions of polysaccharides, proteins and phenolic compounds, all of which can undergo significant changes over the life of the plant [54, 55]. Leaves at different stages may show changes in the intensity and position of the absorption bands also due to the degree of cutinization. These changes may be related to several factors, which are not yet well understood [56], though they may be linked to the formation of new compounds, differences in the formation of hydrogen bonds, changes in crystallinity [57, 58] and differences in the orientation of microfibrils caused by the cell elongation [59].

Throughout their development, plants are cumulatively contaminated by other species, both internally and externally [60, 61, 62]. As a result, a variety of symbionts, parasites and epiphylls may be found in and on plant tissues [63, 64], and these could also modify the spectral signatures. Roberts et al. [65] reported that leaves with a moderate epiphyll coatings and necrosis showed significantly higher NIR absorbance than uncolonized and slightly coated leaves of the same age. The discriminant functions graphs based only on young samples always show greater separation of species than graphs of adults, indicating that some convergence occurs as plants mature, possibly due to shared biotic contaminants.

All these factors could influence the spectral response of species in different developmental stages, but there are no previous studies that confirm this. Also, there are no detailed studies that investigate whether the morphological and chemical changes that occur in the leaves during development do so equally among species [48]. This could explain why some species, such as *Protium paniculatum* var. *riedelianum* seem not to display spectral differences between the stages, while the opposite occurs in *Protium pallidum* (Table 3).

## Conclusion

We conclude that near-infrared spectroscopy has great potential for discriminating species at different developmental stages, and the adult stage spectra can be used to predict the identity of young plants. This opens possibilities for a variety of ecological studies in areas of high plant diversity, where inability to achieve identifications of seedlings for many species has long been a major limiting factor. Hit rates of FT-NIR are comparable to, or higher than, those obtained with DNA barcoding to highly diverse areas such as the tropics, and the technique is much faster, simpler and cheaper. Future studies linking the chemical composition and the spectral pattern of the species across different ontogenetic stages may help understand the origin of these differences and therefore the generality of the patterns we have observed here.
